# Achieving Equity in Transportation for Radiotherapy

**DOI:** 10.7759/cureus.64847

**Published:** 2024-07-18

**Authors:** Tony Song, Karna T Sura

**Affiliations:** 1 Radiation Oncology, State University of New York Upstate Medical University, Syracuse, USA

**Keywords:** s: radiation, health-care equity, radiation oncology payment reform medicare episode case rate model survey, health-care policy, transportation, general radiation oncology

## Abstract

Transportation is a significant social determinant of health and a barrier to treatment for many patients. Cancer patients are disproportionately affected, and it can be especially salient for patients undergoing several weeks of daily radiation treatment. A prospective survey pilot study at our institution examining financial toxicity related to transportation for patients undergoing radiation treatment showed a correlation between high transportation costs and financial stress. Furthermore, those living >10 miles from the radiation center were associated with worse financial toxicity. Previous programs implemented to address the transportation issue in oncology have been mainly inadequate or ineffective. These programs have been set back due to a lack of awareness and low utilization. The Health Equity Achievement in Radiation Therapy (HEART) adjustment from the proposed Radiation Oncology Case Rate (ROCR) payment model for radiation oncology will greatly alleviate transportation barriers for patients undergoing radiation treatment. The $500 per patient can be utilized for those patients at the highest risk, like those living far away from the radiation center.

## Editorial

Transportation has long been a significant social determinant of health and a barrier to treatment for many underserved patients [1]. In many communities, numerous patients in a large geographic area are treated at a single center due to high upfront capital costs limiting the feasibility of opening new facilities [2]. This is especially true for patients living in rural areas where the distance to a treatment center may be more than 50 miles and serves as a larger impediment to the receipt of radiation than in urban areas [2]. Missed appointments can negatively impact patient outcomes due to delays or disruptions in treatment and increased acute care utilization/hospitalization, leading to a 1.2-3.2% absolute increased risk of mortality and representing a significant opportunity cost to our health system [3]. The most disadvantaged populations, including those living in poverty and minority communities, are disproportionately affected [4]. 

Cancer patients, as compared to non-cancer patients, are also disproportionately affected and almost twice as likely to report delays in care due to transportation barriers, according to the National Health Interview Survey [5]. Caretakers of cancer patients are also cognizant of transportation costs, both financially and those associated with opportunity costs, with over half of responders in one study discussing the need for assistance on an institutional or systemic level outside of public transportation, which has been an inadequate solution [6]. Addressing transportation issues not only improves equity and quality of patient care but also reduces medical waste. 

We conducted an IRB-approved prospective pilot survey study examining financial toxicity due to transportation to and from radiation treatment. The Functional Assessment of Chronic Illness Therapy (FACIT) cost measure was licensed and used for this study. Thirty patients completed patient questionnaires, which were collected between August and November 2022 during the COVID-19 pandemic. When gas prices rose over $4 per gallon, the percentage of patients who felt in control of their financial situation significantly decreased (p = 0.049), and the percentage of patients for whom transportation costs associated with radiation treatment became a burden significantly increased (63.2% vs 36.8%, p < 0.001). The high cost of transportation correlated to patient’s feelings of financial stress, dissatisfaction with their current financial situation, and perception of lack of choice in health care spending. Distance to treatment centers >10 miles correlated with the feeling that out-of-pocket medical expenses were higher than expected and patients’ ability to meet monthly expenses. This pilot study shows that transportation costs associated with radiation treatment impact a patient’s financial wellness. Inflation may worsen the impact of this financial toxicity. Although our institution offers financial assistance, a small minority (~10%) knew of the financial assistance, while only 3% utilized financial support for general medical bills and/or travel support. While this pilot study examined only a handful of patients and was limited in part due to the COVID-19 pandemic, larger multi-institutional studies will be needed to validate these findings and strengthen their applicability to diverse healthcare settings. 

Current alternative programs include rideshare programs and larger governmental programs. Data presented at the 2021 American Society of Clinical Oncology (ASCO) Quality Care Symposium by Marquez et al. showed that a rideshare program could drive patient satisfaction and partially offset the cost of missed appointments [7]. Ninety-two percent of patients in this rideshare program indicated that they would not have been able to get to appointments without the program [7]. Similar larger-scale solutions, such as Medicaid’s non-emergency medical transportation (NEMT) system, have emerged, and despite almost $5.5 billion in yearly spending, utilization is low (20% in one study) and has not been shown to reduce the rate of missed appointments [8,9]. This may be explained by the low rate of routine screening approaches used to identify patients with transportation needs. Only a third of National Comprehensive Cancer Network (NCCN) member institutions utilize such routine screening [10]. Our study also demonstrates the lack of awareness of such programs and the need for systematic screening. 

While some institutions have patient financial assistance programs, including gas cards for radiation patients struggling with transportation costs, these programs are limited and poorly funded. A survey of NCCN member institutions found that transportation was predominantly funded by philanthropy, grants, or internal dollars [10]. We believe that the new proposed Radiation Oncology Case Rate (ROCR) payment model will bring a much-needed systematic solution and additional funding for all patients to improve patient access to care. Given the recent Medicare payment trends in radiation oncology with progressive pay cuts to reimbursement amounting to 22% over 10 years, the ASTRO Health Policy Council has proposed the ROCR model to stabilize payments while transitioning to a per-patient payment model. This model would apply to any practice participating in Medicare and would cover both professional and technical services; it is meant to apply to standard techniques but will not cover proton treatment and new technologies. By applying annual inflationary payment updates and savings adjustments, it is expected to limit five-year rebasing declines to no more than 1% and will save Medicare $200 million over five years, or 1% of Medicare spending on radiation oncology.

The Health Equity Achievement in Radiation Therapy (HEART) adjustment from this payment model will address the systemic issue of financial toxicity stemming from transportation costs associated with radiation oncology care and improve equity in quality cancer care. HEART is an adjustment to technical payment, which starts at $500 per patient for transportation assistance for eligible patients to help decrease the burden of transportation for daily treatment. Patients are screened to see if they missed appointments or ADL for the last two months. They would be eligible for a transportation insecurity ICD-10 code (Z59.82) if they have. The radiation center would be given this money through technical components. It is just one of many new features included in the ROCR aimed at improving patient access to care. Importantly, it is a systematic solution that does not require additional screening or resources to implement, is not reliant on external transportation modalities, and is fairly distributed to all patients undergoing radiation treatment. Longitudinal monitoring will be needed to assess the effectiveness of this program and its integration into different healthcare systems. Given cancer patients’ need for transportation support that our study and many others have shown, we hope that HEART adjustment remains an essential component of any payment model being considered.
